# Supplementary material for: Learning genetic perturbation effects with variational causal inference
Source: PLoS Comput Biol. 2026 Feb 2;22(2):e1013194. doi: 10.1371/journal.pcbi.1013194 (PMC12885384; doi:10.1371/journal.pcbi.1013194)
Supplement: S1 Text — (PDF) [file pcbi.1013194.s001.pdf]

A Identifiability

We discuss identifiability of the proposed data-generating process in Fig 1. Identifiability results established in prior theoretical works on causal representation learning [1, 2, 3, 4] are typically guaranteed under specific sets of assumptions. The assumptions most relevant to the application we target here are as follows: (i) unpaired observational and interventional data (corresponding to unpaired control cells and perturbed cells), (ii) interventions that may target multiple latent variables (to reflect genetic perturbations that can directly affect multiple gene modules), and (iii) interventions that are soft, in the sense that they modify the mechanisms of their targeted variables without removing dependencies on their ancestors in the underlying graph. Under these assumptions, results from Jin and Syrgkanis [5] establish that the proposed data-generating process is identifiable up to exact causal graph and a surrounded-node ambiguity over latent causal variables, provided the following additional conditions are satisfied: (1) a linear causal model with distinct noise distributions (Eq. (3) and Assumption 3 in [5]), (2) linear invertible mixing (Assumption 2 in [5]), and (3) non-degeneracy of the causal mechanisms across observational and interventional distributions (Assumption 4 in [5], which requires a sufficiently diverse set of interventions).

B Implementation details

B.1 Architecture and Training

The SCCVAE model is composed of the following:

1. *Expression encoder*: After the input cell expressions are log normalized, they are passed through 2 fully connected hidden layers of size 1024. Mean and variance encoders are fully connected layers of output size 512 that accept the output of the shared encoding. Each intermediate layer uses a leaky ReLU activation function with negative slope 0.2.

2. *Shift encoder*: 1 fully connected hidden layer of size 1024, followed by Leaky ReLU activation (negative slope=0.2) and an output layer of size 512. Output is softmaxed.

3. *SCM*: The causal graph is simulated using a size 512 $\times$ 512 parameter initialized to a Gaussian distribution with mean 0 and variance 0.1, multiplied by an upper triangular binary mask. Shift values are initialized as a 1 $\times$ 512 model parameter.

4. *Expression decoder*: 2 fully connected hidden layers of size 1024, followed by an output layer of size 8563. All layers except for output use LeakyReLU with negative slope 0.2.

The model was trained using a learning rate of 5e-4, a linear KL annealing schedule with $\beta_{max} = 1$, and MMD scale $\gamma = 10$. To represent the perturbation p, the top 50 PCA components were used. To stabilize training, a reconstruction term for the control distribution is added, where $U^{\varnothing}$ is decoded with $c^p = 0$ to reconstruct $X^{\varnothing}$. This term is scaled by 0.25 for in-distribution splits and out-of-distribution splits 0, 2, 3, and 4, and scaled by 1 for out-of-distribution split 1. Inclusion of this term reduces the risk of exploding gradients, but does not significantly affect numerical results.

B.2 Hyperparameter Selection

Hyperparameter selection for learning rate, β, γ, and neural network size were performed using a grid search and selected based on lowest validation error according to the validation sets described in Section 3.1. Hyperparameters were selected from the following lists of possible values:

Learning Rate	[1e-3, 5e-4, 1e-4]
β	[1, 2, 4, 8]
γ	[1, 2, 4, 8, 10, 15]
X reconstruction term	[0.0, 0.25, 0.5, 0.75, 1.0]
Number of layers in encoder/decoder	[1, 2, 3]
Layer size	[512, 1024]

B.3 Shift Selection

After training, when presented with an unseen perturbation, we search for an optimal shift value within the range of c^p learned by the model. Using increments of 0.1, we grid search every possible shift value within range. For each shift value, we substitute for c^q and compute $\hat{X}^q$. The final value of c^q is set to the value that minimizes $MSE(X^q, \hat{X}^q)$, comparing to the mean expression value obtained from bulk data for perturbation q. Since we do not have access to an alterative bulk screen, we utilize pseudo-bulk expressions computed from the same dataset.

B.4 Quantitative Metrics

B.4.1 Mean squared error.

The mean squared error (MSE) metric evaluates the error between the means of the predicted and ground-truth distributions:

$$\mathrm{MSE}(X^p, \hat{X}^p) = \mathbb{E}[(\mathbb{E}[X^p] - \mathbb{E}[\hat{X}^p])^2].$$

The final value reported is averaged across all genes of the mean normalized expression level.

B.4.2 Pearson correlation.

We also evaluate the Pearson correlation between the distribution means of expression changes from control, using a similar approach to the evaluation of MSE:

$$\mathrm{PearsonR}(X^p, \hat{X}^p) = \frac{\mathrm{cov}(\mathbb{E}[X^p] - \mathbb{E}[X], \mathbb{E}[\hat{X}^p] - \mathbb{E}[X])}{\sigma_{\mathbb{E}[X^p]-\mathbb{E}[X]}\sigma_{\mathbb{E}[\hat{X}^p]-\mathbb{E}[X]}}$$

where X is the control cell distribution, $\mathrm{cov}(\cdot)$ is the covariance and $\sigma_{(\cdot)}$ is the standard deviation of each mean expression value.

B.4.3 Maximum Mean Discrepancy.

The maximum mean discrepancy (MMD) evaluates a distribution level error:

$$\text{MMD}(X^p, \hat{X}^p) = \|\mathbb{E}_{x^p \sim X^p}[\phi(x^p)] - \mathbb{E}_{\hat{x}^p \sim \hat{X}^p}[\phi(\hat{x}^p)]\|_2$$

where $\phi(\cdot)$ is the Gaussian kernel function.

B.4.4 Energy Distance.

To compute energy distance (D^2), we compare the prediction and ground truth on a distributional level as follows:

$$D^2(X^p, \hat{X}^p) = 2\mathbb{E}[d(X^p, \hat{X}^p)] - \mathbb{E}[d(X^p, X_1^p)] - \mathbb{E}[d(\hat{X}^p, \hat{X}_1^p)]$$

where X_1^p and $\hat{X}_1^p$ are independent samples from the same distributions as X^p and $\hat{X}^p$ respectively (implemented as random permutations of cells). The distance function is given by $d(X, Y) = \mathbb{E}[(X - Y)^2]$.

To correct for the effect of distribution sample size on error magnitude, MMD and Energy distance were computed in batches of size 32 averaged across the entire test dataset.

B.4.5 Direction of changes.

We evaluate the quality of the model predictions on gene differential expression. For each perturbation, we compare the fraction of genes where the direction of change from the control cell distribution is the same as that of the ground truth (Fraction same), as well as the fraction of genes that are changed in the opposite direction (Fraction changed):

$$\begin{aligned}
\Delta(X^p, \hat{X}^p) &= |(\mathbb{E}[X^p] - \mathbb{E}[X]) - (\mathbb{E}[\hat{X}^p] - \mathbb{E}[X])| \\
\text{Fraction_same}(X^p, \hat{X}^p) &= Pr[\Delta(X^p, \hat{X}^p) = 0] \\
\text{Fraction_changed}(X^p, \hat{X}^p) &= Pr[\Delta(X^p, \hat{X}^p) = 2]
\end{aligned}$$

where X is the control control cell distribution.

C Additional Results

C.1 Subset of Perturbations

Fig A in S1 Text shows the process for selecting perturbations for training. From the starting set of perturbations, approximately 20% are perturbations with 200 cells or more. Out of these, around 68% have a logistic regression score of 0.6 or more. Then, we remove perturbations that have no identified causal relationships with other observed genes, or are not included in the expression vector. This leaves 279 perturbational distributions for our analysis.

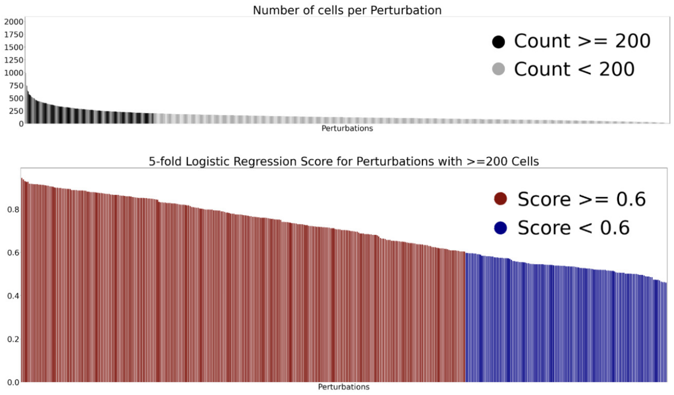

Fig A in S1 Text: We limit our analysis to perturbations with over 200 single cells. Logistic regression filtering for perturbational distributions on the remaining perturbations. Only perturbations wtih a 5-fold cross validation logistic regression score of 0.6 or higher are considered.

C.2 In-distribution Results

Table A in S1 Text shows the results of SCCVAE training on the in-distribution data set. It can be seen that SCCVAE beats both GEARS and control baselines for all metrics, both on the entire set of essential genes and on the top 50 most highly variable genes in the control data set. In Table B in S1 Text, we observe similar trends to the OOD results in that the original SCCVAE model outperforms the other versions of the model in MSE and MMD metrics. However, we note here that the Causal-GSP model does not outperform the conditional model (except for energy distance), unlike in the OOD task, and the conditional model is also able to outperform the original SCCVAE on the energy distance metric. This is because the ability to utilize a causal graph and generalize to new cells is less crucial for the in-distribution task, since the test cells are from the same distribution as train cells.

C.3 Breakdown of Distance Scatterplots by Training/testing Distributions

Fig B in S1 Text looks at the correlation between MMD and latent space distance (similar to Fig 4) for train and test perturbations separately. There is a strong positive correlation in training test perturbations, as expected, but the test set perturbations also exhibit positive correlations across all five splits.

C.4 Additional Shift Selection Results

Fig C in S1 Text shows the same procedure as Fig 5 repeated on a different gene, NLE1. For NLE1, the post-perturbational expression distribution is much closer to the control distribution, and the shift value that yield the lowest error is also much smaller in magnitude ($c^p \approx 0.5$). We can see that as c^p become larger, the predicted distribution also moves farther away from the ground truth.

**All genes**	**Control**	**GEARS**	**SCCVAE**
MSE	$0.0131_{\pm 0.00641}$	$0.0118_{\pm 0.00568}$	$\mathbf{0.0114_{\pm 0.004}}$
PearsonR	$-0.000_{\pm 0.047}$	$0.112_{\pm 0.058}$	$\mathbf{0.321_{\pm 0.186}}$
MMD	$0.242_{\pm 0.0431}$	$0.239_{\pm 0.0440}$	$\mathbf{0.220_{\pm 0.01}}$
Energy Distance	$0.0363_{\pm 0.0200}$	$0.0325_{\pm 0.0176}$	$\mathbf{0.0296_{\pm 0.01}}$
Fraction Same	$0.497_{\pm 0.0127}$	$0.541_{\pm 0.0319}$	$\mathbf{0.587_{\pm 0.04}}$
Fraction Changed	$0.471_{\pm 0.0127}$	$0.459_{\pm 0.0319}$	$\mathbf{0.413_{\pm 0.04}}$

**Top 50 genes**	**Control**	**GEARS**	**SCCVAE**
MSE	$0.0153_{\pm 0.00970}$	$0.0134_{\pm 0.00846}$	$\mathbf{0.0126_{\pm 0.006}}$
PearsonR	$-0.005_{\pm 0.159}$	$0.136_{\pm 0.164}$	$\mathbf{0.325_{\pm 0.228}}$
MMD	$0.249_{\pm 0.0602}$	$0.244_{\pm 0.0583}$	$\mathbf{0.227_{\pm 0.03}}$
Energy Distance	$0.0425_{\pm 0.0336}$	$0.0370_{\pm 0.0317}$	$\mathbf{0.0316_{\pm 0.02}}$
Fraction Same	$0.501_{\pm 0.0589}$	$0.548_{\pm 0.0762}$	$\mathbf{0.586_{\pm 0.08}}$
Fraction Changed	$0.459_{\pm 0.0589}$	$0.452_{\pm 0.0762}$	$\mathbf{0.414_{\pm 0.08}}$

Table A in S1 Text: In-distribution results for SCCVAE compared to GEARS and the control baseline, for all essential genes and for top 50 highly variable genes, averaged across all perturbational distributions.

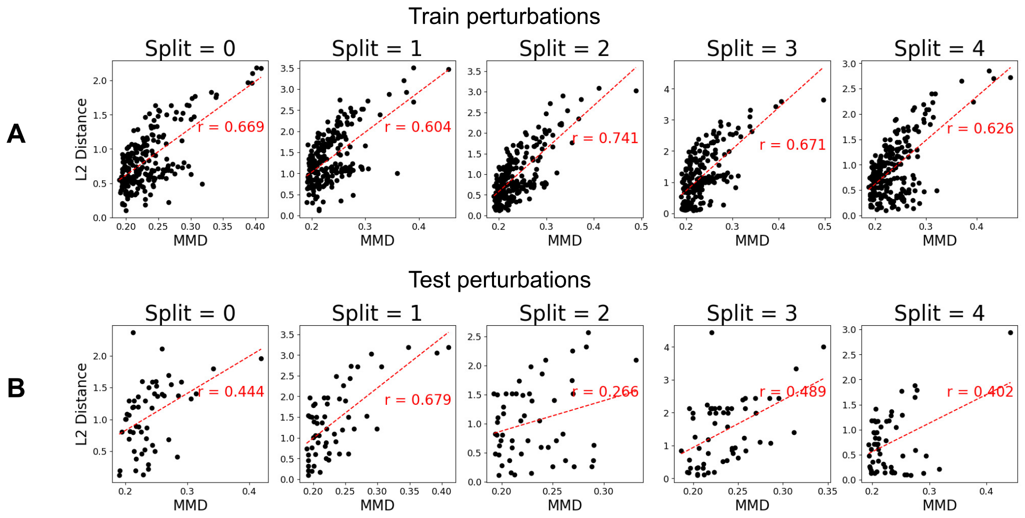

Fig B in S1 Text: Continuation of Fig 4. (A) In just the training/validation set perturbations, the latent variables are learned directly during training (since no shift selection is required). This leads to stronger correlations overall. (B) In the test set, latent variables are computed after selecting shift to minimize MSE. Although the correlation is weaker than the training set perturbations, it is consistently positive across all splits.

All genes	SCCVAE	Conditional	Causal-GSP	Random
MSE	$\mathbf{0.0114}_{\pm\mathbf{0.00439}}$	$0.0117_{\pm 0.00473}$	$0.0117_{\pm 0.00426}$	$0.0155_{\pm 0.00396}$
PearsonR	$0.321_{\pm 0.186}$	$0.285_{\pm 0.196}$	$0.320_{\pm 0.179}$	$\mathbf{0.397}_{\pm\mathbf{0.131}}$
MMD	$\mathbf{0.220}_{\pm\mathbf{0.0180}}$	$0.221_{\pm 0.0220}$	$0.221_{\pm 0.0220}$	$0.276_{\pm 0.0234}$
Energy Dist.	$0.0296_{\pm 0.0183}$	$0.0287_{\pm 0.0182}$	$\mathbf{0.0266}_{\pm\mathbf{0.0160}}$	$0.0354_{\pm 0.0110}$
Frac. Same	$0.587_{\pm 0.0475}$	$0.575_{\pm 0.0497}$	$0.579_{\pm 0.0473}$	$\mathbf{0.599}_{\pm\mathbf{0.0385}}$
Frac. Changed	$0.413_{\pm 0.0476}$	$0.425_{\pm 0.0497}$	$0.421_{\pm 0.0473}$	$\mathbf{0.401}_{\pm\mathbf{0.0385}}$

Top 50 genes	SCCVAE	Conditional	Causal-GSP	Random
MSE	$\mathbf{0.0126}_{\pm\mathbf{0.00692}}$	$0.0129_{\pm 0.00692}$	$0.0130_{\pm 0.00687}$	$0.0192_{\pm 0.00643}$
PearsonR	$0.325_{\pm 0.228}$	$0.308_{\pm 0.231}$	$0.318_{\pm 0.230}$	$\mathbf{0.397}_{\pm\mathbf{0.184}}$
MMD	$\mathbf{0.227}_{\pm\mathbf{0.0339}}$	$0.229_{\pm 0.0360}$	$0.228_{\pm 0.0375}$	$0.304_{\pm 0.0400}$
Energy Dist.	$0.0316_{\pm 0.0293}$	$\mathbf{0.0312}_{\pm\mathbf{0.0278}}$	$0.0352_{\pm 0.0270}$	$0.0438_{\pm 0.0203}$
Frac. Same	$0.586_{\pm 0.0815}$	$0.577_{\pm 0.0899}$	$0.581_{\pm 0.0813}$	$\mathbf{0.602}_{\pm\mathbf{0.06}}$
Frac. Changed	$0.414_{\pm 0.0815}$	$0.423_{\pm 0.0899}$	$0.419_{\pm 0.0813}$	$\mathbf{0.398}_{\pm\mathbf{0.06}}$

Table B in S1 Text: Ablation studies on the in-distribution task. Unlike in the OOD task, the conditional and causal GSP models achieve better performance than the original SCCVAE for the energy distance metric.

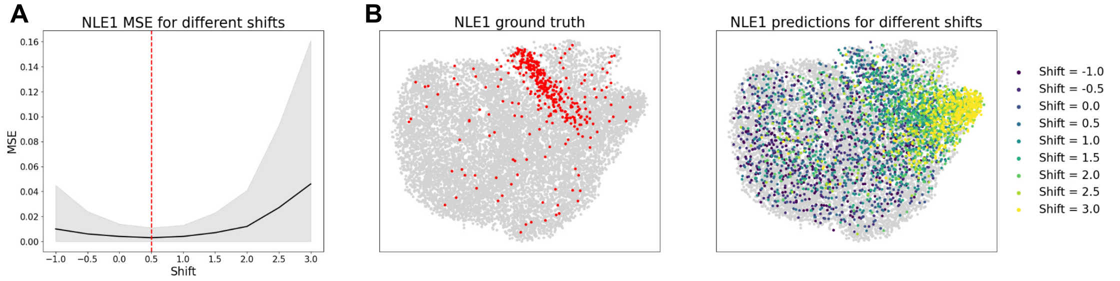

Fig C in S1 Text: (A) Shift selection for a different perturbation (NLE1), where the optimal shift value is closer to control. (B) When c^p is increased, we can visualize the postperturbational cell expression distribution under a higher intensity gene knockdown.

C.5 Additional Perturbation Modules

Fig D in S1 Text visualizes the embeddings of 12 different functional perturbation modules in U^p latent space. As stated already, the embeddings of genes within one perturbational module form a cluster in the latent space. We observe that even across different perturbation modules affecting the same cellular functions, similar functional modules are also grouped closely in the latent space. For example, all genes encoding ribosomal proteins or ribosome biogenesis are close to each other, chromatin and DNA controlling genes are grouped closely together, and splicing genes are also grouped near each other.

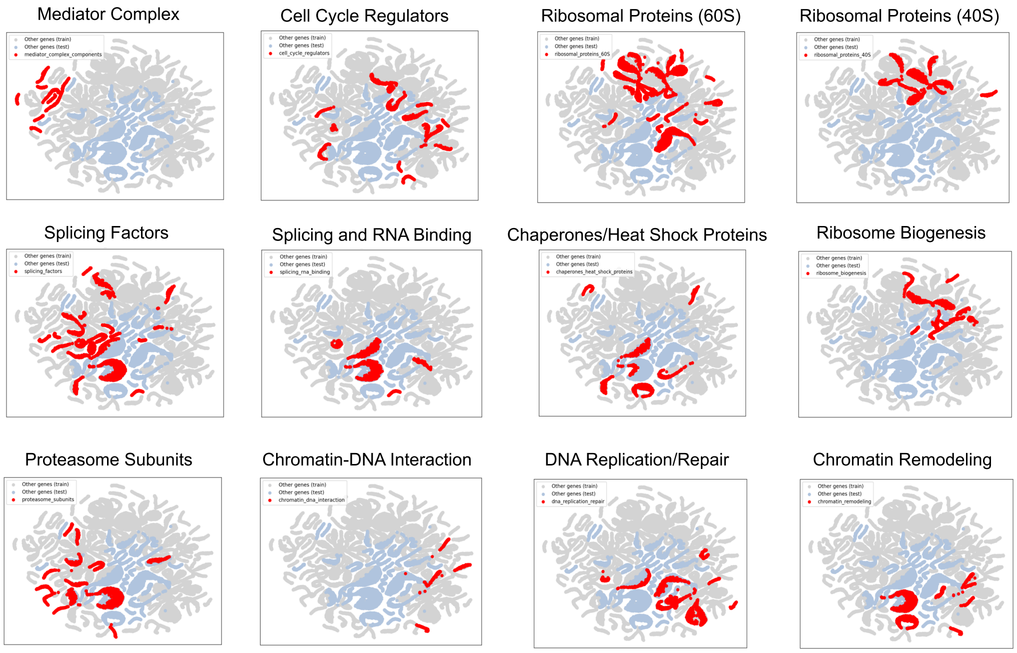

Fig D in S1 Text: Individual perturbation modules. In each figure, the red regions indicate U^p embeddings of cells that are part of the perturbation module, the grey regions indicate U^p embeddings of non-module cells from the training set, and the blue regions indicate U^q embeddings of non-module cells from the test set.

C.6 Interpretation of the Learned SCM

Fig E in S1 Text visualizes the structural causal model learned by SCCVAE (Section 2.1) in the in-distribution experiment on $N = 279$ perturbations on K562 cells. As the learned graph $\mathcal{G}$ is encoded by the adjacency matrix $A \in \mathbb{R}^{512\times512}$, we first use A to read out a learned network. The perturbations alter variables in this network by the encoded shift vectors $S = (S^{p_1}, \ldots, S^{p_{279}}) \in \mathbb{R}^{279\times512}$.

To interpret the learned network, we cluster the dimensions of this network according to their responses to the perturbations. In particular, we use K-means clustering based on $S^\top$ with 11 clusters. We then aggregate the adjacency matrix A by averaging the scores between dimensions in each pair of clusters. A cluster is considered to be more upstream than a distinct cluster if in less than half of the entries in the sub-matrix of A corresponding these two clusters are zero. By re-orienting the clusters from upstream to downstream, we visualize the aggregated absolute connections in this network in Fig E in S1 Text.

To interpret how each perturbation S^p alters the individual clusters in the aggregated network, we average the entries of S^p corresponding to each cluster and use the averaged vector as the scoring for this perturbation. Based on the averaged scoring vector, we visualize 10 random perturbations per cluster in Fig E in S1 Text. Notably cluster 0 is mainly contributed by the Ribosome family. A deeper understanding of these clusters may reveal biological insights.

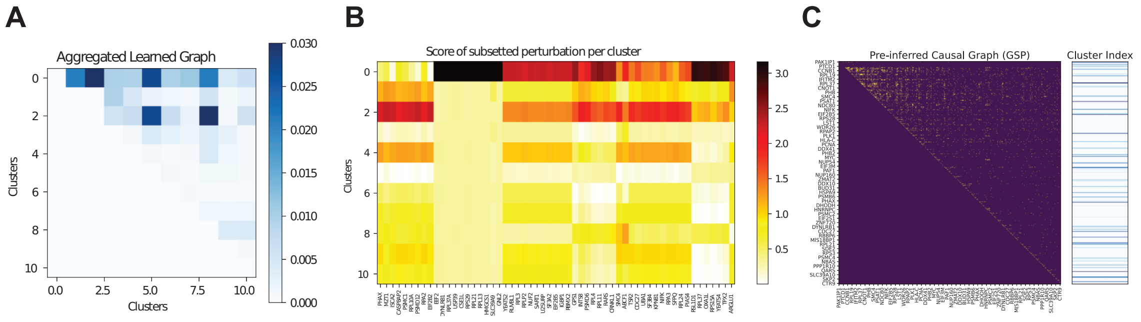

Fig E in S1 Text: SCM Learned by SCCVAE. (A) Learned adjacency matrix A aggregated by clusters found using the encoded shift vectors S. (B) Learned shift vectors S averaged using the clusters found in (A) on a subset of perturbations. (C) Causal graph pre-inferred by GSP, annotated with the subset of perturbations shown in (B). Connections in the causal graph are highlighted in yellow within the heatmap, where the graph is visualized after topological sorting. The annotation bar indicates the perturbations from (B), with colors denoting the cluster index in which each perturbation appears from left to right.

We additionally compared the learned SCM to the pre-specified causal graph inferred by GSP, shown in Fig E in S1 Text. We annotated the entries in the GSP-inferred graph with the 10 perturbations per cluster from Fig E in S1 Text. Overall, we observed that the GSP-inferred graph is distinct from the learned SCM. Nevertheless, some perturbations that appear in the same clusters in the learned graph also tend to group together in the GSP-inferred graph.

C.7 Comparison Across Datasets

We demonstrate the capacity of SCCVAE over other datasets. Out of the considered baselines, GEARS has shown to have consistent performances across metrics, therefore we compare against GEARS for analysis over other datasets. In Table C in S1 Text, we compare SCCVAE to GEARS and control cell baselines on the entire dataset, without selecting for high signal-to-noise ratio perturbational distributions. Here, we observe that SCCVAE still generally out-performs baselines, although the difference is less dramatic than in the selected perturbations case. Additionally, both GEARS and control baselines appear to achieve lower error on the full dataset compared to the selected dataset, whereas SCCVAE exhibits the opposite trend and achieves higher error on the full dataset. The most likely explanation for this is that the full dataset contains many perturbational distributions that are very similar to the control distribution. As a result, the control baselines are lower, and GEARS, which outputs similar predictions to control, achieves lower error. SCCVAE, which tends to identify differences from the control distribution, generally performs better when the perturbed distribution is distinct from the control distribution.

**All genes**	**Control**	**GEARS**	**SCCVAE**
MSE	$0.0077_{\pm 0.0077}$	$0.00731_{\pm 0.00745}$	$\mathbf{0.00657_{\pm 0.004}}$
PearsonR	$0.000112_{\pm 0.0476}$	$0.0758_{\pm 0.0653}$	$\mathbf{0.140_{\pm 0.152}}$
MMD	$0.213_{\pm 0.0286}$	$\mathbf{0.212_{\pm 0.0308}}$	$0.217_{\pm 0.02}$
Energy Distance	$0.0272_{\pm 0.0138}$	$0.0264_{\pm 0.0136}$	$\mathbf{0.0262_{\pm 0.01}}$
Fraction Same	$0.484_{\pm 0.0067}$	$0.522_{\pm 0.0226}$	$\mathbf{0.544_{\pm 0.04}}$
Fraction Changed	$0.483_{\pm 0.0067}$	$0.478_{\pm 0.0226}$	$\mathbf{0.456_{\pm 0.04}}$

**Top 50 genes**	**Control**	**GEARS**	**SCCVAE**
MSE	$0.00861_{\pm 0.00962}$	$0.00809_{\pm 0.0093}$	$\mathbf{0.00739_{\pm 0.006}}$
PearsonR	$-0.00127_{\pm 0.166}$	$0.0866_{\pm 0.172}$	$\mathbf{0.139_{\pm 0.219}}$
MMD	$0.215_{\pm 0.0446}$	$\mathbf{0.214_{\pm 0.044}}$	$0.221_{\pm 0.03}$
Energy Distance	$0.0291_{\pm 0.0243}$	$\mathbf{0.0279_{\pm 0.0237}}$	$0.0283_{\pm 0.0}$
Fraction Same	$0.52_{\pm 0.0722}$	$0.528_{\pm 0.0744}$	$\mathbf{0.548_{\pm 0.08}}$
Fraction Changed	$0.48_{\pm 0.0722}$	$0.472_{\pm 0.0744}$	$\mathbf{0.452_{\pm 0.08}}$

Table C in S1 Text: Evaluation on the entire K562 single-cell dataset, without selecting for high signal-to-noise ratio perturbations. Since GEARS exhibited the best performance out of the two model baselines, we evaluate against GEARS and the control baseline. SCCVAE achieves lower error than control or GEARS for all metrics except MMD with all essential genes and all metrics except MMD and Energy Distance with top 50 genes. Compared to evaluation on the selected perturbation dataset, evaluation on all genes appears to be an easier task for all models. This is likely due to the similarity of most distributions to control, as evidenced by the fact that GEARS exhibits a larger drop in performance on the selected dataset than SCCVAE.

In Table E in S1 Text, we evaluate SCCVAE on the dataset from Norman et al., which targets a smaller set of genes on the K562 cell line compared to the Replogle et al. dataset ($n = 105$). On both the full set of essential genes and the top 50 most variable genes, SCCVAE achieves the lowest mean squared error (MSE) and highest Pearson correlation, indicating more accurate prediction of perturbation-induced changes. It also outperforms both baselines on distributional alignment metrics such as MMD and energy distance when evaluated on all genes, suggesting a stronger match to the full single-cell expression distribution. While GEARS achieves a slightly better MMD than SCCVAE on the top 50 genes, this comes at the cost of lower accuracy in MSE and Pearson correlation. Directional agreement metrics (fraction of genes with same or changed direction from control) show comparable performance between SCCVAE and GEARS, though SCCVAE shows greater stability across gene subsets.

Table D in S1 Text shows results upon evaluating a selected RPE1 dataset, also from Replogle et al. [6]. While SCCVAE continues to outperform GEARS and control cell baselines on most metrics when evaluating on all essential genes, both GEARS and SCCVAE consistently do not beat the control cell baseline when only the top 50 highly variable genes are considered, with SCCVAE usually achieving lower error than GEARS. There are two possible causes for this phenomenon. First, the RPE1 dataset is smaller than the K562 dataset, and the neural network based methods may require

more data than is available to learn optimally. Second, the RPE1 cell line is non-cancerous, while the K562 cell line is cancerous, meaning that they derive from fundamentally different distributions. It is possible that these models are more performant on cancerous cell lines, as they may better capture the underlying biological variability and gene expression patterns that are more pronounced in cancerous cells due to their altered regulatory mechanisms and higher levels of genetic instability. This suggests that these models might implicitly exploit features or patterns specific to cancer biology, leading to diminished performance on non-cancerous cell lines like RPE1, which exhibit more stable and less variable gene expression profiles. Future work could explore tailoring these models to account for differences in cell line characteristics or incorporating stronger priors to improve generalization across multiple different cell lines.

**All genes**	**Control**	**GEARS**	**SCCVAE**
MSE	$0.00715_{\pm 0.00782}$	$0.00635_{\pm 0.00704}$	$\mathbf{0.00634_{\pm 0.00618}}$
PearsonR	$-0.0287_{\pm 0.123}$	$-0.00589_{\pm 0.0764}$	$\mathbf{0.288_{\pm 0.246}}$
MMD	$0.237_{\pm 0.0558}$	$\mathbf{0.233_{\pm 0.0511}}$	$0.239_{\pm 0.0446}$
Energy Distance	$0.0305_{\pm 0.0146}$	$0.03_{\pm 0.0142}$	$\mathbf{0.0299_{\pm 0.0134}}$
Fraction Same	$0.485_{\pm 0.00948}$	$0.503_{\pm 0.023}$	$\mathbf{0.545_{\pm 0.0524}}$
Fraction Changed	$0.485_{\pm 0.00948}$	$0.497_{\pm 0.023}$	$\mathbf{0.455_{\pm 0.0524}}$

**Top 50 genes**	**Control**	**GEARS**	**SCCVAE**
MSE	$\mathbf{0.0092_{\pm 0.0113}}$	$0.0122_{\pm 0.0253}$	$0.0121_{\pm 0.0238}$
PearsonR	$-0.128_{\pm 0.373}$	$-0.0758_{\pm 0.33}$	$\mathbf{0.400_{\pm 0.362}}$
MMD	$\mathbf{0.249_{\pm 0.074}}$	$0.265_{\pm 0.144}$	$0.277_{\pm 0.124}$
Energy Distance	$\mathbf{0.0343_{\pm 0.0248}}$	$0.0406_{\pm 0.0497}$	$0.04_{\pm 0.0498}$
Fraction Same	$0.485_{\pm 0.0756}$	$0.507_{\pm 0.075}$	$\mathbf{0.546_{\pm 0.0935}}$
Fraction Changed	$0.495_{\pm 0.0756}$	$0.493_{\pm 0.075}$	$\mathbf{0.454_{\pm 0.0935}}$

Table D in S1 Text: Evaluation on the RPE1 cell line. SCCVAE achieves lower error than baselines when considering all essential genes, but the control cell baseline has lower error than both GEARS and SCCVAE when considering the top 50 highly variable genes.

All genes	Control	GEARS	SCCVAE
MSE	$0.00539_{\pm 0.00590}$	$0.00448_{\pm 0.00515}$	$\mathbf{0.00329_{\pm 0.000743}}$
PearsonR	$-0.00178_{\pm 0.121}$	$0.00282_{\pm 0.104}$	$\mathbf{0.419_{\pm 0.0498}}$
MMD	$0.327_{\pm 0.121}$	$0.307_{\pm 0.100}$	$\mathbf{0.273_{\pm 0.0114}}$
Energy Distance	$0.0159_{\pm 0.0127}$	$0.0139_{\pm 0.0105}$	$\mathbf{0.0117_{\pm 0.00136}}$
Fraction Same	$0.335_{\pm 0.00659}$	$\mathbf{0.566_{\pm 0.0314}}$	$0.536_{\pm 0.00452}$
Fraction Changed	$\mathbf{0.301_{\pm 0.0067}}$	$0.413_{\pm 0.0324}$	$0.442_{\pm 0.00466}$

Top 50 genes	Control	GEARS	SCCVAE
MSE	$0.00675_{\pm 0.0109}$	$0.00678_{\pm 0.0154}$	$\mathbf{0.00564_{\pm 0.00118}}$
PearsonR	$-0.0472_{\pm 0.234}$	$-0.0573_{\pm 0.271}$	$\mathbf{0.348_{\pm 0.0431}}$
MMD	$0.299_{\pm 0.165}$	$\mathbf{0.285_{\pm 0.159}}$	$0.308_{\pm 0.0203}$
Energy Distance	$0.0203_{\pm 0.0228}$	$0.0220_{\pm 0.0306}$	$\mathbf{0.0185_{\pm 0.00199}}$
Fraction Same	$0.403_{\pm 0.0605}$	$0.555_{\pm 0.0733}$	$\mathbf{0.557_{\pm 0.0129}}$
Fraction Changed	$\mathbf{0.357_{\pm 0.0602}}$	$0.445_{\pm 0.0733}$	$0.442_{\pm 0.0129}$

Table E in S1 Text: Comparison of SCCVAE against baseline models on the Norman et al. dataset. SCCVAE demonstrates improved predictive accuracy and distributional alignment across most metrics, particularly on global gene expression patterns.

All genes	SCCVAE	MMD_Loss = 0
MSE	$\mathbf{0.00542_{\pm 0.00295}}$	$0.0062_{\pm 0.00448}$
PearsonR	$\mathbf{0.498_{\pm 0.164}}$	$0.490_{\pm 0.143}$
MMD	$\mathbf{0.229_{\pm 0.0193}}$	$2.10_{\pm 0.055}$
Energy Dist.	$0.0299_{\pm 0.00958}$	$\mathbf{0.0226_{\pm 0.0104}}$
Frac. Same	$\mathbf{0.630_{\pm 0.0509}}$	$0.626_{\pm 0.070}$
Frac. Changed	$\mathbf{0.370_{\pm 0.0509}}$	$0.374_{\pm 0.070}$
**Top 50 genes**	**SCCVAE**	**MMD_Loss = 0**
MSE	$\mathbf{0.00651_{\pm 0.00537}}$	$0.00816_{\pm 0.00734}$
PearsonR	$\mathbf{0.500_{\pm 0.205}}$	$0.439_{\pm 0.234}$
MMD	$\mathbf{0.236_{\pm 0.0323}}$	$2.02_{\pm 0.0776}$
Energy Dist.	$0.0338_{\pm 0.0160}$	$\mathbf{0.0266_{\pm 0.0163}}$
Frac. Same	$0.612_{\pm 0.082}$	$\mathbf{0.618_{\pm 0.0975}}$
Frac. Changed	$0.388_{\pm 0.0821}$	$\mathbf{0.381_{\pm 0.0975}}$

Table F in S1 Text: Ablation study assessing the impact of removing the MMD loss term from SCCVAE. Excluding MMD results in a substantial degradation in distributional alignment (MMD), with minor drops in prediction accuracy (MSE, PearsonR), especially for the most variable genes.

C.8 Loss Function Ablation

To assess the contribution of the MMD loss term in the SCCVAE objective, we conduct an ablation study by training a variant of SCCVAE with the MMD loss coefficient set to zero. As shown in Table F in S1 Text, removing the MMD loss substantially degrades performance on the MMD metric itself, as expected, with the score increasing nearly tenfold. This indicates a severe mismatch between predicted and ground truth single-cell distributions.

Notably, while the Energy Distance slightly improves without MMD regularization, this improvement is modest and may reflect the less penalized optimization of marginal moment matching. In contrast, metrics related to prediction accuracy (MSE and PearsonR) consistently degrade when the MMD loss is removed, particularly for the top 50 most variable genes.

These results suggest that the MMD loss is critical in not only in improving the fidelity of predicted distributions but also in regularizing the model to enhance overall generalization performance. The directional agreement metrics (Fraction of genes with same or changed direction) remain largely unaffected, indicating that while coarse directionality may be preserved, the fine-grained distributional structure is substantially impaired without the MMD term.

References

[1] Jiaqi Zhang, Kristjan Greenewald, Chandler Squires, Akash Srivastava, Karthikeyan Shanmugam, and Caroline Uhler. Identifiability guarantees for causal disentanglement from soft interventions. *Advances in Neural Information Processing Systems*, 36:50254–50292, 2023.

[2] Julius von Kügelgen, Michel Besserve, Liang Wendong, Luigi Gresele, Armin Kekić, Elias Bareinboim, David Blei, and Bernhard Schölkopf. Nonparametric identifiability of causal representations from unknown interventions. *Advances in Neural Information Processing Systems*, 36:48603–48638, 2023.

[3] Burak Varıcı, Emre Acartürk, Karthikeyan Shanmugam, Abhishek Kumar, and Ali Tajer. Score-based causal representation learning: Linear and general transformations. *arXiv preprint arXiv:2402.00849*, 2024.

[4] Simon Bing, Urmi Ninad, Jonas Wahl, and Jakob Runge. Identifying linearly-mixed causal representations from multi-node interventions. In *Causal Learning and Reasoning*, pages 843–867. PMLR, 2024.

[5] Jikai Jin and Vasilis Syrgkanis. Learning causal representations from general environments: Identifiability and intrinsic ambiguity. *arXiv preprint arXiv:2311.12267*, 2023.

[6] Joseph M Replogle, Reuben A Saunders, Angela N Pogson, Jeffrey A Hussmann, Alexander Lenail, Alina Guna, Lauren Mascibroda, Eric J Wagner, Karen Adelman, Gila Lithwick-Yanai, et al. Mapping information-rich genotype-phenotype landscapes with genome-scale perturb-seq. *Cell*, 185(14):2559–2575, 2022.
