# Supplementary material for: Learning genetic perturbation effects with variational causal inference
Source: PLoS Comput Biol. 2026 Feb 2;22(2):e1013194. doi: 10.1371/journal.pcbi.1013194 (PMC12885384; doi:10.1371/journal.pcbi.1013194)
Supplement: Attachment — Submitted filename: report.pdf [file pcbi.1013194.s002.pdf]

Referee Report for PCOMPBIOL-D-25-01122

The manuscript by Emily Liu, Jiaqi Zhang, and Caroline Uhler describes a mechanistic-informed AI model called SCCVAE for inferring the underlying gene regulatory network from single-cell Perturb-Seq datasets. Through simulation studies and real data analyses, it was claimed that SCCVAE exhibits superior performance in terms of predicting the consequence of unseen perturbations. Overall, the paper contributes to the data analysis of Perturb-Seq data, one of the most promising data types for rigorously drawing causal conclusions in molecular biology.

This paper essentially instantiates the idea of causal disentanglement or causal representation learning (CRL) to single-cell biology. CRL is a quite recent direction in the causal machine learning literature. But related concepts have appeared in the classical statistical literature under the broad umbrella of factor analysis, independent component analysis, and/or sufficient dimension reduction. CRL can be a daunting subject for biologists or even computational biologists to comprehend. I personally find that the current exposition style of the paper makes it even worse, due to lack of explanation or logical leaps at several points. I also could not fully appreciate why using CRL in this specific context makes a difference. This is especially so after looking into the experimental results that do not demonstrate a clear advantage over other more standard techniques. I also have the following more detailed comments/suggestions that I hope the authors could find useful.

Major comments:

1. Identification is at the core of causality. It is no exception for CRL. I understand that this paper targets computational biologists. However, at least after reading the paper, it is not clear to me what unique aspect causality brings. Is the proposed model at all identifiable? There has been some progress on identifiability analysis of CRL recently, some of which are also produced by the two corresponding authors (and also groups led by Varici or Syrgkanis). It might be worth discussing the identifiability issue, or at the very minimum, referencing some papers supporting identifiability.
2. It is also unclear about the benefit of using the CRL framework instead of the more traditional causal discovery framework, the goal of which is to discover causality among observables. Is the reason scientific or computational (dimension reduction from m to n)? By scientific, I mean that whether it is well accepted prior knowledge that gene modules rather than individual genes are the fundamental units for causality.
3. The latent representation output by SCCVAE is interpreted as gene modules, an abstraction of individual genes into functional units. Could the authors provide more biological evidence about the learnt modules? For instance, I would imagine that different modules might share the same genes. Do those genes have any special features?
4. In the manuscript, I find that there is a lack of connection to the data. For example, on page 4, it was mentioned that "multivariate Gaussian distributions, which we observe to sufficiently fit the transcriptomic data." This observation was served as the basis of the Gaussian structural causal model imposed on the latent gene modules. But there seems to be a logical leap – the observed transcription data shall generally be non-Gaussian. It will be better to refer to some empirical evidence to support such a claim, which is quite central to the new method.

5. I find the choice of $n = 512$ quite mysterious. In a scientific paper, it is fine to set certain tuning parameters to a specific value, based on either model fit or some biological reasons. Maybe it does not really matter to choose a different value or maybe it does. But I could not draw a conclusion myself based on the evidence given in the paper.
6. The reason for using the top PC computed from the control cell matrix as the perturbation label should be further elucidated. It is not immediately obvious to me why this is a reasonable choice.
7. When describing the results, there is an extremely confusing sentence "Out of the remaining perturbations, we consider only the perturbations that yield an average five-fold cross validation score of greater than 0.6 in a logistic regression task distinguishing a perturbed cell from negative controls". I highly recommend that this sentence be re-organized.
8. It might be helpful to explain in the caption of Figure 2 why no information on MMD for linear models. It was explained in the text that linear models are not readily able to handle distribution learning but it was after describing the results in Figure 2.
9. Based on the experimental results (such as Figure 2 and Table 1), it is not obvious (at least to me) the advantage of the new method. I can only say that the new method is not worse than existing ones. Then what is the significance of bringing causality into the picture? Concepts developed in the past decades in causal inference are mainly designed to help researchers think deeply about assumptions and identifications (such as works by Eric Tchetgen Tchetgen and colleagues). But this type of thinking does not seem to appear in this work. More specifically, I think I am simply looking for a concrete example where

computational biologists may easily make a mistake without thinking about causality but may avoid such a mistake by incorporating causality.

Minor comments:

1. In the abstract, it was mentioned that “While deep learning models excel at interpolating observed perturbational data, they tend to overfit and may not generalize well to unseen perturbations.” This point may happen in certain applications but not necessarily so without providing further contexts. I recommend that a weaker statement be presented, such as “they tend to overfit in the lack of big data”.
2. On page 2: “For example, Kamimoto et al. [24], Zhang et al. [25] uses a linear causal model based on a learned gene regulatory network”. It is recommended that the two works be discussed separately.
3. On page 4, cyclic causal graphs were brought up. Some references on cyclic causal graphs are recommended to be cited as readers might be interested in learning about related work, given the prevalence of feedback loop in the gene regulatory process and the conceptual difficulty of handling cycles.
4. Variational lower bound is used in the paper. It seems that a more common nomenclature in variational inference is “evidence lower bound”.
5. In the beginning of Section 3: “We evaluate **SCCVAE** on the single-gene perturbational datasets”.
6. The beginning of page 7: “The additional experimental results can be found in Appendix A.3.6**.**”
