# Supplementary material for: Learning genetic perturbation effects with variational causal inference
Source: PLoS Comput Biol. 2026 Feb 2;22(2):e1013194. doi: 10.1371/journal.pcbi.1013194 (PMC12885384; doi:10.1371/journal.pcbi.1013194)
Supplement: Attachment — Submitted filename: Response to Reviewers.pdf [file pcbi.1013194.s003.pdf]

Response to Reviewer #1

The manuscript by Emily Liu, Jiaqi Zhang, and Caroline Uhler describes a mechanistic-informed AI model called SCCVAE for inferring the underlying gene regulatory network from single-cell Perturb-Seq datasets. Through simulation studies and real data analyses, it was claimed that SCCVAE exhibits superior performance in terms of predicting the consequence of unseen perturbations. Overall, the paper contributes to the data analysis of Perturb-Seq data, one of the most promising data types for rigorously drawing causal conclusions in molecular biology. This paper essentially instantiates the idea of causal disentanglement or causal representation learning (CRL) to single-cell biology. CRL is a quite recent direction in the causal machine learning literature. But related concepts have appeared in the classical statistical literature under the broad umbrella of factor analysis, independent component analysis, and/or sufficient dimension reduction. CRL can be a daunting subject for biologists or even computational biologists to comprehend. I personally find that the current exposition style of the paper makes it even worse, due to lack of explanation or logical leaps at several points. I also could not fully appreciate why using CRL in this specific context makes a difference. This is especially so after looking into the experimental results that do not demonstrate a clear advantage over other more standard techniques. I also have the following more detailed comments/suggestions that I hope the authors could find useful.

We thank the reviewer for their thoughtful evaluation and for recognizing the significance of our contribution to Perturb-seq data analysis. We address each of the points raised in our point-by-point response below. Revisions made in response to the reviewer's comments are highlighted in red in the revised manuscript.

Major comments

1. Identification is at the core of causality. It is no exception for CRL. I understand that this paper targets computational biologists. However, at least after reading the paper, it is not clear to me what unique aspect causality brings. Is the proposed model at all identifiable? There has been some progress on identifiability analysis of CRL recently, some of which are also produced by the two corresponding authors (and also groups led by Varici or Syrgkanis). It might be worth discussing the identifiability issue, or at the very minimum, referencing some papers supporting identifiability.

We thank the reviewer for this question and suggestion. Identifiability results established in prior theoretical work on CRL are typically guaranteed under specific sets of assumptions. The assumptions most relevant to the application we target in this manuscript are as follows: (i) unpaired observational and interventional data (corresponding to unpaired control cells and perturbed cells), (ii) interventions that may target multiple latent variables (to reflect genetic

perturbations that can directly affect multiple pathways), and (iii) interventions that are soft, in the sense that they modify the mechanisms of their targeted variables without removing dependencies on their ancestors in the underlying graph.

Our earlier theoretical work (Zhang et al., 2023a), together with many recent studies on single-node or hard interventions (e.g., Varici et al., 2025; Bing et al., 2024), does not fully address this setting, as these works do not account for assumptions (ii) or (iii).

Under the assumptions described above, results from Jin and Syrgkanis (2023) establish that the proposed data-generating process is identifiable up to exact causal graph and a surrounded-node ambiguity over latent causal variables, provided the following additional conditions are satisfied: (1) a linear causal model with distinct noise distributions (Eq. (3) and Assumption 3 in Jin and Syrgkanis, 2023), (2) linear invertible mixing (Assumption 2 in Jin and Syrgkanis, 2023), and (3) non-degeneracy of the causal mechanisms across observational and interventional distributions (Assumption 4 in Jin and Syrgkanis, 2023, which requires a sufficiently diverse set of interventions).

We have added this discussion to Appendix A: "We discuss identifiability of the proposed … which requires a sufficiently diverse set of interventions).". We also included a corresponding pointer as a footnote in Section 2.1.2 of the revised manuscript.

2. It is also unclear about the benefit of using the CRL framework instead of the more traditional causal discovery framework, the goal of which is to discover causality among observables. Is the reason scientific or computational (dimension reduction from m to n)? By scientific, I mean that whether it is well accepted prior knowledge that gene modules rather than individual genes are the fundamental units for causality.

We thank the reviewer for this question. We would like to address it together with the related question about the unique aspects of causality raised in the previous point. The main contribution of this work is an algorithmic method that combines mechanistic modeling with scalable deep variational approaches. The motivation is twofold, as the reviewer noted.

First, in our previous work (Zhang et al., 2023b), we observed challenges in fitting and generalization to novel perturbations when applying traditional causal discovery frameworks, as the number of perturbations and genes increases. In that study, we considered a relatively small setting with only 36 genes, where it was feasible to fit a linear causal model and this model is helpful for designing effective interventions. However, in larger datasets such as the one considered in this paper—with the largest experimental setting containing 8,000 perturbations and over 8,000 genes—the traditional framework requires substantial improvement.

Second, in modeling perturbation effects, it has remained an open question what constitutes the appropriate representation of the fundamental units of causality. We hypothesize that such a representation should be data-efficient, in the sense that it captures the underlying biological

mechanisms. This naturally motivates grouping genes into modules, a strategy already widely used in biological research to understand gene function (Subramanian et al., 2005 PMID: 16199517). If this modular representation indeed proves to be more data-efficient, then it also provides a more suitable way to represent causality in this setting.

3. The latent representation output by SCCVAE is interpreted as gene modules, an abstraction of individual genes into functional units. Could the authors provide more biological evidence about the learnt modules? For instance, I would imagine that different modules might share the same genes. Do those genes have any special features?

We thank the reviewer for this question. We interpret the latent representations in two complementary ways.

First, at a coarser level, we examine how these latent representations vary across perturbations. This corresponds to analyzing the distribution of latent causal variables under different interventions. As shown in Fig 6 (Section 3.4) and Fig 10 (Appendix C.5), these distributions differ across perturbations but cluster according to perturbations targeting known complexes. In this way, the latent representations provide a reliable basis for defining groups of perturbations with similar functions, including cell-cycle regulators, DNA replication and repair factors, and the mediator complex.

Second, along with what the reviewer suggested, we also analyze the latent representations at the level of individual dimensions. Here, each dimension is interpreted as a gene module. By examining which perturbations directly alter each dimension relative to the control, we can infer both the composition of each gene module (i.e., which genes belong to it) and the relationships between different gene modules. This analysis is presented in Appendix C.6. There, we find that certain gene modules correspond to sets of genes with well-established functions, and in particular, that the top-ranked modules are primarily composed of ribosomal genes.

We additionally compared the learned SCM to the pre-specified causal graph inferred by GSP. This result is added to Appendix C.6 in the revised manuscript and attached below. As the learned graph groups genes into modules, whereas GSP returns a graph between individual genes, we aim to see if genes grouped in the same modules were close to each other the graph learned by GSP. Therefore we annotated the entries in the GSP-inferred graph with the 10 perturbations per cluster from the learned graph. Overall, we observed that the GSP-inferred graph is distinct from the learned SCM. Nevertheless, some perturbations that appear in the same clusters in the learned graph also tend to group together in the GSP-inferred graph.

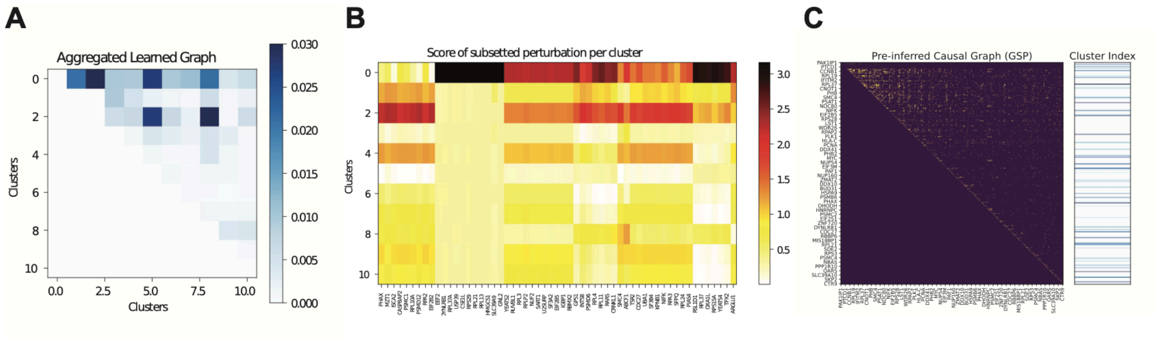

Figure 11: SCM Learned by SCCVAE. (A) Learned adjacency matrix A aggregated by clusters found using the encoded shift vectors S. (B) Learned shift vectors S averaged using the clusters found in (A) on a subset of perturbations. (C) Causal graph pre-inferred by GSP, annotated with the subset of perturbations shown in (B). Connections in the causal graph are highlighted in yellow within the heatmap, where the graph is visualized after topological sorting. The annotation bar indicates the perturbations from (B), with colors denoting the cluster index in which each perturbation appears from left to right.

4. In the manuscript, I find that there is a lack of connection to the data. For example, on page 4, it was mentioned that "multivariate Gaussian distributions, which we observe to sufficiently fit the transcriptomic data." This observation was served as the basis of the Gaussian structural causal model imposed on the latent gene modules. But there seems to be a logical leap – the observed transcription data shall generally be non-Gaussian. It will be better to refer to some empirical evidence to support such a claim, which is quite central to the new method.

We thank the reviewer for this suggestion. It is the case that raw single-cell transcriptomic data are counts and non-Gaussian. Prior literature (c.f., Gayoso et al., 2022) has approached this in two main ways: either by modeling transcriptomic data with (zero-inflated) negative binomial distributions, or by applying normalization, log-transformation, and potential dimensionality reduction to enable Gaussian modeling (which is the approach taken in the present manuscript). These methods are referring to modeling directly in the observed transcriptomic space.

The claim we made quoted by the reviewer refers to the latent space, which is linked to the observed transcriptomic space through a decoder that can flexibly transform a latent Gaussian distribution into the appropriate family of distributions in the output space. In our analysis, we find that the latent representations can be well-approximated by multivariate Gaussian distributions. This observation has also been empirically supported in prior work using Gaussian-based latent modeling of single-cell data (Lopez et al., 2018, Lotfollahi et al., 2020). In our setting, the latent embeddings U^p produced by SCCVAE exhibit approximately Gaussian structure after fitting, as reflected in the improved fit and performance of a Gaussian structural causal model in this space. This motivates our choice of a linear Gaussian SCM to model the relationships among gene modules in the latent representation space.

We have added this explanation and reference to Section 2.1.1: “Here the Gaussian family is … single-cell data [29, 30].”

5. I find the choice of n = 512 quite mysterious. In a scientific paper, it is fine to set certain tuning parameters to a specific value, based on either model fit or some biological reasons. Maybe it does not really matter to choose a different value or maybe it does. But I could not draw a conclusion myself based on the evidence given in the paper.

We chose $n = 512$ as it provided good model fit during training and validation. We expect that the results would not change substantially if n were varied within a reasonable range, although some hyperparameters such as the learning rate might need to be adjusted accordingly. We specified $n = 512$ in the main text to make the model setup more transparent, but this detail is not essential to our main findings. For clarity, we have changed it into a footnote in the revised manuscript.

6. The reason for using the top PC computed from the control cell matrix as the perturbation label should be further elucidated. It is not immediately obvious to me why this is a reasonable choice.

As the task is to generalize to unseen perturbations, we required a perturbation label (i.e., the input to the perturbation encoder) that could (i) relate unseen perturbations to those observed during training, and (ii) be computable for perturbations without available perturbational data. The rationale for using the principal components of the transposed control cell matrix is that it satisfies condition (ii), since it does not rely on perturbational data. Moreover, it produces similar representations for genes that co-express in control cells. This choice reflects the inductive bias that genes with similar functions—and therefore likely to induce similar perturbational effects—tend to co-express under natural variations such as the heterogeneity observed in control cells. A similar prior has also been adopted in previous work (Ahlmann-Eltze et al., 2024).

We have added this explanation to Section 2.1.2 of the revised manuscript: “ This produces similar representations for genes that co-express … adopted in previous work [20].”

7. When describing the results, there is an extremely confusing sentence “Out of the remaining perturbations, we consider only the perturbations that yield an average five-fold cross validation score of greater than 0.6 in a logistic regression task distinguishing a perturbed cell from negative controls”. I highly recommend that this sentence be reorganized.

Thank you for this comment. We have revised the text to: "Out of the perturbations with sufficient cells, we consider the subset that is distinct from controls and thus requires nontrivial predictions. This subset is identified by performing a logistic regression task to distinguish

perturbed cells from negative controls. Perturbations achieving an average five-fold cross-validation score greater than 0.6 are retained, resulting in $N = 279$ perturbations."

8. It might be helpful to explain in the caption of Figure 2 why no information on MMD for linear models. It was explained in the text that linear models are not readily able to handle distribution learning but it was after describing the results in Figure 2.

Thank you for this suggestion. We have added the explanation to the caption of Figure 2 in the revised manuscript.

9. Based on the experimental results (such as Figure 2 and Table 1), it is not obvious (at least to me) the advantage of the new method. I can only say that the new method is not worse than existing ones. Then what is the significance of bringing causality into the picture? Concepts developed in the past decades in causal inference are mainly designed to help researchers think deeply about assumptions and identifications (such as works by Eric Tchetgen Tchetgen and colleagues). But this type of thinking does not seem to appear in this work. More specifically, I think I am simply looking for a concrete example where 3 computational biologists may easily make a mistake without thinking about causality but may avoid such a mistake by incorporating causality.

Thank you for this question. The goal of this paper is not to address scenarios where inference mistakes can easily appear without thinking about causality (as in works by Eric Tchetgen Tchetgen and colleagues that the reviewer mentioned). Rather, our aim is to explore how mechanistic or causal models can be used in a data-efficient manner to model interventions, particularly in the context of high-dimensional readouts and a large number of perturbations. As noted in our response to a previous question, traditional causal models struggle to fit and generalize effectively in this setting, which motivated our investigation into combining mechanistic models with scalable deep learning approaches.

We believe that constraining scalable approaches with mechanistic models could be a promising direction for improving perturbation modeling, especially when high-fidelity prior causal networks are available. In preliminary experiments, we attempted to enforce the latent causal structure to match a previously learned network (section 5.2), which performed slightly worse than the fully learning-based approach. Notably, the learning-based approach performs on par with—and in some cases better than—current baselines, suggesting its potential as an off-the-shelf framework for incorporating mechanistic models, whether fully learned, enforced, or warm-started. As technologies advance and higher-quality data become available, we expect this approach to become increasingly valuable.

Minor comments:

1. In the abstract, it was mentioned that “While deep learning models excel at interpolating observed perturbational data, they tend to overfit and may not generalize well to unseen perturbations.” This point may happen in certain applications but not necessarily so without providing further contexts. I recommend that a weaker statement be presented, such as “they tend to overfit in the lack of big data”.

Thank you for this suggestion. We have revised accordingly.

2. On page 2: “For example, Kamimoto et al. [24], Zhang et al. [25] uses a linear causal model based on a learned gene regulatory network”. It is recommended that the two works be discussed separately.

Thank you for this suggestion. We have revised the text accordingly: "Kamimoto et al. [24] used a linear model based on a pre-fixed gene regulatory network inferred from ChIP-seq data and demonstrated its utility for recommending transcription factors for direct differentiation. Zhang et al. [25] employed a linear causal model based on a learned gene regulatory network obtained via causal discovery algorithms and showed its usefulness for intervention design in a semi-synthetic experiment."

3. On page 4, cyclic causal graphs were brought up. Some references on cyclic causal graphs are recommended to be cited as readers might be interested in learning about related work, given the prevalence of feedback loop in the gene regulatory process and the conceptual difficulty of handling cycles.

Thank you for this suggestion. We have added additional pointers on cyclic causal graphs: “Extensions of acyclic causal models to cyclic models can be considered to incorporate feedback loops. When the causal variables are observed, methods for causal discovery in acyclic models have been extended to cyclic settings, including constraint-based algorithms (e.g., Richardson, 2013, Mooij and Claassen, 2020, Strobl, 2019) which generalize conditional independence tests from d-separation to σ-separation, and score-based algorithms (e.g., Lacerda et al., 2012, Mooij et al., 2011).”

4. Variational lower bound is used in the paper. It seems that a more common nomenclature in variational inference is “evidence lower bound”.
5. In the beginning of Section 3: “We evaluate SCCVAE on the single-gene perturbational datasets”.
6. The beginning of page 7: “The additional experimental results can be found in Appendix A.3.6.”

Thank you for the additional comments above. We have revised the manuscript accordingly.

References

Zhang, J., Greenewald, K., Squires, C., Srivastava, A., Shanmugam, K., & Uhler, C. (2023). Identifiability guarantees for causal disentanglement from soft interventions. *Advances in Neural Information Processing Systems*, *36*, 50254-50292.

Varici, B., Acartürk, E., Shanmugam, K., Kumar, A., & Tajer, A. (2025). Score-based causal representation learning: Linear and general transformations. *Journal of Machine Learning Research*, *26*(112), 1-90.

Bing, S., Ninad, U., Wahl, J., & Runge, J. (2024, March). Identifying linearly-mixed causal representations from multi-node interventions. In *Causal Learning and Reasoning* (pp. 843-867). PMLR.

Jin, J., & Syrgkanis, V. (2023). Learning causal representations from general environments: Identifiability and intrinsic ambiguity. *arXiv preprint arXiv:2311.12267*.

Zhang, J., Cammarata, L., Squires, C., Sapsis, T. P., & Uhler, C. (2023). Active learning for optimal intervention design in causal models. *Nature Machine Intelligence*, *5*(10), 1066-1075.

Subramanian, A., Tamayo, P., Mootha, V. K., Mukherjee, S., Ebert, B. L., Gillette, M. A., ... & Mesirov, J. P. (2005). Gene set enrichment analysis: a knowledge-based approach for interpreting genome-wide expression profiles. Proceedings of the National Academy of Sciences, 102(43), 15545-15550.

Gayoso, A., Lopez, R., Xing, G., Boyeau, P., Valiollah Pour Amiri, V., Hong, J., ... & Yosef, N. (2022). A Python library for probabilistic analysis of single-cell omics data. *Nature biotechnology*, *40*(2), 163-166.

Lopez, R., Regier, J., Cole, M. B., Jordan, M. I., & Yosef, N. (2018). Deep generative modeling for single-cell transcriptomics. *Nature methods*, *15*(12), 1053-1058.

Lotfollahi, M., Wolf, F. A., & Theis, F. J. (2019). scGen predicts single-cell perturbation responses. *Nature methods*, *16*(8), 715-721.

Ahlmann-Eltze, C., Huber, W., & Anders, S. (2024). Deep learning-based predictions of gene perturbation effects do not yet outperform simple linear baselines. *BioRxiv*, 2024-09.

Richardson, T. S. (2013). A discovery algorithm for directed cyclic graphs. arXiv preprint arXiv:1302.3599.

Mooij, J. M., & Claassen, T. (2020, August). Constraint-based causal discovery using partial ancestral graphs in the presence of cycles. In Conference on Uncertainty in Artificial Intelligence (pp. 1159-1168). PMLR.

Strobl, E. V. (2019). A constraint-based algorithm for causal discovery with cycles, latent variables and selection bias. International Journal of Data Science and Analytics, 8(1), 33-56.

Lacerda, G., Spirtes, P. L., Ramsey, J., & Hoyer, P. O. (2012). Discovering cyclic causal models by independent components analysis. arXiv preprint arXiv:1206.3273.

Mooij, J. M., Janzing, D., Heskes, T., & Schölkopf, B. (2011). On causal discovery with cyclic additive noise models. Advances in neural information processing systems, 24.

Response to Reviewer #2

In this manuscript, the authors present SCCVAE, a deep generative causal inference framework designed to uncover the regulatory network underlying single-cell genetic perturbations. SCCVAE models single-cell gene expression using a structural causal model, where the intrinsic gene modules U_i are assumed to regulate each other through a linear relationship. In the experiments, the authors demonstrated that SCCVAE outperforms baselines like GEARS and scGPT in prediction of single-cell level perturbation effects. Overall the paper is well written, and the proposed method is very interesting. However, there are some questions for the authors to address for publication.

We thank the reviewer for recognizing the significance of our work and for their careful evaluation. We address the points raised in the reviewer's comments in the point-by-point response below. All edits made in response to these comments are highlighted in red in the revised manuscript.

1. (testing on datasets with fewer target genes) In the manuscript, the authors primarily demonstrate the effectiveness of SCCVAE using cell line data with a large number of target genes from Replogle et al. (2022). I wonder how SCCVAE performs when the number of target genes is smaller in the training data, for example, fewer than one hundred?

Thank you for this question. Currently, the smallest experiment used by Replogle et al. involves 280 perturbations. We additionally evaluated our method on the Norman dataset, which includes approximately 105 single-gene perturbations targeting a smaller set of genes in the K562 cell line ($n = 105$). These results are reported in Table 7 and attached below.

Across both the full set of essential genes and the top 50 most variable genes, SCCVAE achieves the lowest mean squared error (MSE) and the highest Pearson correlation, indicating more accurate prediction of perturbation-induced changes. SCCVAE also outperforms both baselines on distributional alignment metrics, such as MMD and energy distance, when evaluated across all genes, suggesting a stronger match to the full single-cell expression distribution. While GEARS attains a slightly better MMD on the top 50 genes, this comes at the cost of lower accuracy in terms of MSE and Pearson correlation. Directional agreement metrics, which measure the fraction of genes with the same or altered direction relative to control, show comparable performance between SCCVAE and GEARS, though SCCVAE demonstrates greater stability across gene subsets.

**All genes**	**Control**	**GEARS**	**SCCVAE**
MSE	0.00539±0.00590	0.00448±0.00515	**0.00329±0.000743**

PearsonR (change)	−0.00178±0.121	0.00282±0.104	**0.419±0.0498**
MMD	0.327±0.121	0.307±0.100	**0.273±0.0114**
Energy Distance	0.0159±0.0127	0.0139±0.0105	**0.0117±0.00136**
Fraction Same	0.335±0.00659	**0.566±0.0314**	0.536±0.00452
Fraction Changed	**0.301±0.0067**	0.413±0.0324	0.442±0.00466

**Top 50 genes**	**Control**	**GEARS**	**SCCVAE**
MSE	0.00675±0.0109	0.00678±0.0154	**0.00564±0.00118**
PearsonR (change)	−0.0472±0.234	−0.0573±0.271	**0.348±0.0431**
MMD	0.299±0.165	**0.285±0.159**	0.308±0.0203
Energy Distance	0.0203±0.0228	0.0220±0.0306	**0.0185±0.00199**
Fraction Same	0.403±0.0605	0.555±0.0733	**0.557±0.0129**
Fraction Changed	**0.357±0.0602**	0.445±0.0733	0.442±0.0129

Table 7: Comparison of SCCVAE against baseline models on the Norman et al. dataset. SCCVAE demonstrates improved predictive accuracy and distributional alignment across most metrics, particularly on global gene expression patterns.

2. (evaluation) For the comparison, I suggest using the Pearson correlation between predicted and true gene expression changes, instead of between expressions. This is because the correlation between the control cell and perturbed cell gene expression is already very close to 1.

Thank you for this suggestion. We have revised the Pearson correlation results to reflect the differences between control and perturbed cells (see Table 7 above and Tables 1–6). We observed that this reflects a bigger difference between different methods and that the comparison between different methods—both regarding the comparison of SCCVAE to baseline methods and the ablation studies—remains consistent.

3. (model training) SCCVAE incorporates an additional MMD term during training to align distributions. It would be helpful to provide more details on the implementation of this term (such

as how many samples are used for its estimation during training and the choice of kernels) and to demonstrate its contribution to the prediction performance through a simple ablation study.

Thank you for this suggestion. We have added the ablation results in Supplementary Appendix C.8. In particular, to assess the contribution of the MMD loss term in the SCCVAE objective, we conduct an ablation study by training a variant of SCCVAE with the MMD loss coefficient set to zero. As shown in Table 8 (attached below), removing the MMD loss substantially degrades performance on the MMD metric itself, as expected, with the score increasing nearly tenfold. This indicates a severe mismatch between predicted and ground truth single-cell distributions.

Notably, while the Energy Distance slightly improves without MMD regularization, this improvement is modest and may reflect the less penalized optimization of marginal moment matching. In contrast, metrics related to prediction accuracy (MSE and PearsonR) consistently degrade when the MMD loss is removed, particularly for the top 50 most variable genes. These results suggest that the MMD loss is critical not only in improving the fidelity of predicted distributions but also in regularizing the model to enhance overall generalization performance. The directional agreement metrics (Fraction of genes with same or changed direction) remain largely unaffected, indicating that while coarse directionality may be preserved, the fine-grained distributional structure is substantially impaired without the MMD term.

**All genes**	**SCCVAE**	**MMD_Loss = 0**
MSE	**0.00542±0.00295**	0.0062±0.00448
PearsonR (change)	**0.498±0.164**	0.490±0.143
MMD	**0.229±0.0193**	2.10±0.055
Energy Distance	0.0299±0.00958	**0.0226±0.0104**
Fraction Same	**0.630±0.0509**	0.626±0.070
Fraction Changed	**0.370±0.050**	0.374±0.070

**Top 50 genes**	**SCCVAE**	**MMD_Loss = 0**
MSE	**0.00651±0.00537**	0.00816±0.00734
PearsonR (change)	**0.500±0.205**	0.439±0.234
MMD	**0.236±0.0323**	2.02±0.0776
Energy Distance	0.0338±0.0160	**0.0266±0.0163**

Fraction Same	0.612±0.082	**0.618±0.0975**
Fraction Changed	0.388±0.0821	**0.381±0.0975**

Table 8: Ablation study assessing the impact of removing the MMD loss term from SCCVAE. Excluding MMD results in a substantial degradation in distributional alignment (MMD), with minor drops in prediction accuracy (MSE, PearsonR), especially for the most variable genes.

4. (model interpretability) The authors present the interpretability of the learned SCM in the supplementary notes. However, this is a key strength of SCCVAE that sets it apart from many other perturbation prediction models, and it should be highlighted in the main text. Additionally, it would be interesting to explore differences between the learned graph and a pre-inferred graph using methods like Causal-GSP, beyond just comparing prediction accuracy.

Thank you for this suggestion. We have revised the manuscript by highlighting this component in section 3.4 “Interpretability of SCCVAE”. In addition, we added an analysis comparing the learned graph with a pre-inferred graph in Appendix C.6.

As the learned graph groups genes into modules, whereas GSP returns a graph between individual genes, we aim to see if genes grouped in the same modules were close to each other the graph learned by GSP. Therefore we annotated the entries in the GSP-inferred graph with the 10 perturbations per cluster from the learned graph. Overall, we observed that the GSP-inferred graph is distinct from the learned SCM. Nevertheless, some perturbations that appear in the same clusters in the learned graph also tend to group together in the GSP-inferred graph.

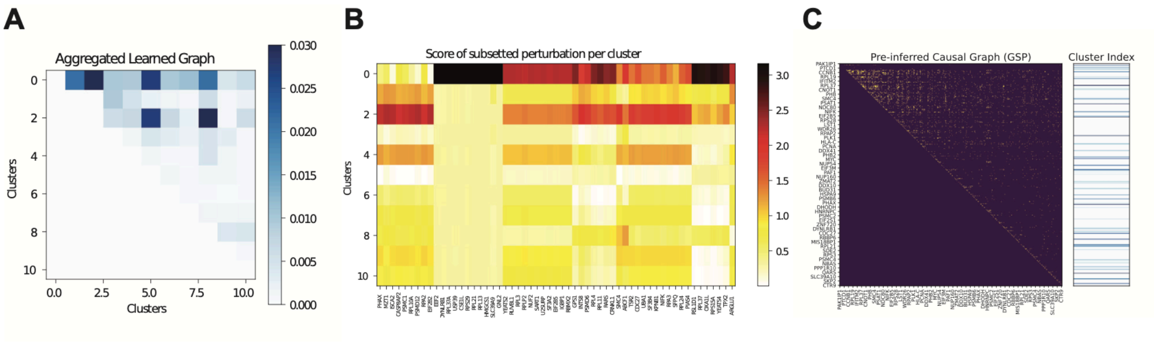

Figure 11: SCM Learned by SCCVAE. (A) Learned adjacency matrix A aggregated by clusters found using the encoded shift vectors S. (B) Learned shift vectors S averaged using the clusters found in (A) on a subset of perturbations. (C) Causal graph pre-inferred by GSP, annotated with the subset of perturbations shown in (B). Connections in the causal graph are highlighted in yellow within the heatmap, where the graph is visualized after topological sorting. The annotation bar indicates the perturbations from (B), with colors denoting the cluster index in which each perturbation appears from left to right.
