# Supplementary material for: Learning genetic perturbation effects with variational causal inference
Source: PLoS Comput Biol. 2026 Feb 2;22(2):e1013194. doi: 10.1371/journal.pcbi.1013194 (PMC12885384; doi:10.1371/journal.pcbi.1013194)
Supplement: Attachment — Submitted filename: SCCVAE Plos Comp Bio Response to Reviewers.pdf [file pcbi.1013194.s004.pdf]

# Response to Reviewer #1

The authors' thorough rebuttal and revision have addressed my main concerns. I have only one very minor suggestion. There is a recently published work also building causality into modern AI-type models (diffusion models) with the hope to get better generalization in comp-bio problems: https://www.nature.com/articles/s41467-025-63728-0. It is recommended to also briefly discuss this work, in terms of the differences in focus, assumptions, and settings.

We thank the reviewer for their time and are pleased that the rebuttal and revisions have addressed their main concerns. We have addressed the remaining suggestion by adding the following discussion of CausCell to the Introduction:

"*Gao et al. proposes to model single-cell transcriptomics data using a diffusion model with cross-attentions that incorporate disentangled concept embeddings. The concept embeddings are constrained by a predefined causal graph that encodes the causal relationships between concepts (e.g., tissues, cell types, perturbations), which enables counterfactual generation upon changing these concepts. Note that this causal graph differs from that of gene regulation described above, as it models the relationships between hierarchical concepts*."

## Response to Reviewer #2

In their response, the authors have addressed most of my concerns and substantially clarified the paper. However, based on the revised results, one issue remains confusing: the use of random graphs often achieves better PearsonR, which is counterintuitive. This suggests that a misspecified causal structure among gene modules might somehow improve perturbation effect prediction. It works better on a subset of pseudo-bulk level metrics (table 2), but not distributional level metrics (table 2 & fig 3). Same for in-distribution & out-of-distribution.

We are pleased that our previous response and revisions have addressed most of the reviewer's concerns and improved the clarity of the paper. We are happy to provide the requested additional clarification. The random-graph version performs well, compared to the learned graph, on a subset of pseudo-bulk–level metrics that are easier: they primarily capture the dominant signal of the data. Both Pearson correlation and fraction-correct (the pseudo-bulk metric that random-graph version works well) reflect directional changes relative to control; they do not require the predicted numerical values to match the ground truth closely, only that the relative direction of change is correct (as PearsonR computes correlation between predicted and ground-truth change vectors).

We think that for these easier metrics, the random-graph version performs quite well, and in some cases better than the learned-graph version, because it belongs to a simpler model class. Intuitively, when the graph structure is fixed to be random rather than learned, the model has fewer degrees of freedom. This effectively constrains the model class, making the "easier" pseudo-bulk metrics less challenging to predict. Additional evidence that simpler model classes perform well on these metrics comes from the linear baseline (see Table 1), which also achieves strong results on this subset.

We added this discussion to Section 3.3.3: "*It is worth noting that the subset of pseudo-bulk metrics that the random graph model is able to perform well on are metrics that do not require the predicted numerical values, but only the relative directional change, to match the ground truth closely. The random-graph model imposes a stronger sparsity constraint on the latent causal graph compared to the learned-graph model, which may make these metrics easier to predict. As shown in Table 1, a simple linear model performs quite well on these metrics. However, when the mechanisms in the model are mis-specified (e.g., with a random graph), it is unable to correct the prediction for MSE and distributional metrics*."

I also noticed that, in the ablation studies, the DAG was learned without sparsity constraints, whereas in the main method the authors reported that using an upper-triangular mask worked well. The authors should explain why the DAG structures are inconsistent and clarify how different choices of DAG parameterizations contribute to improving the model.

We thank the reviewer for this comment. In the ablation studies, the DAG is learned with an upper-triangular mask, consistent with the main method. In our original sentence in section 3.3 ("*The results in Table 1 are generated using a learned DAG without sparsity restrictions.*"), the "sparsity restrictions" was referring to the additional sparsity restrictions on the lower-triangular part (i.e., non masked part), as adopted by the causal-GSP and random graph variants. We apologize for this confusion, and have revised the sentence to "*The results in Table 1 are generated using a learned DAG with additional sparsity restrictions (i.e., only upper-triangular mask is applied)*".

The different variants in the ablation studies (Table 2) can be interpreted as different choices of DAG parameterizations, with varying sparsity constraints:

- The conditional variant is equivalent to applying no masking at all on the DAG.
- The SCCVAE (with a learned graph) masks only the upper-triangular part of the DAG.
- The causal-GSP and random variants apply additional masking: beyond the upper-triangular part, they also mask entries in the lower-triangular part that correspond to missing edges. In these cases, causal-GSP uses a graph pre-learned from observational data, whereas the random variant uses a randomly generated graph.

We have added this discussion to Section 3.3: "*Note that these different choices reflect varying sparsity constraints: the conditional variant is equivalent to applying no masking on the DAG; the SCCVAE (with a learned graph) masks only the upper-triangular part of the DAG; the causal-GSP and random variants apply additional masking: beyond the upper-triangular part, they also mask entries in the lower-triangular part that correspond to missing edges. In these cases, causal-GSP uses a graph pre-learned from observational data, whereas the random variant uses a randomly generated graph*."

Finally, in Section 3.3.3, the statement "The error metrics (MSE, Pearson, MMD, Energy Distance) for the random graph model are significantly worse than the other models" should be revised considering the updated results.

Thank you for pointing this out! We have revised it accordingly.
